# Novel slag/natural rubber composite as flexible material for protecting workers against radiation hazards

**DOI:** 10.1038/s41598-023-40846-7

**Published:** 2023-08-22

**Authors:** Ahmed M. El-Khatib, A. S. Doma, Mahmoud I. Abbas, Abd El-Hady B. Kashyout, Mohamed M. Zaki, Moamen Saleh, Mahmoud T. Alabsy

**Affiliations:** 1 Physics Department, Faculty of Science, Alexandria University, Alexandria, 21511 Egypt; 2 Advanced Technology and New Materials Research Institute (ATNMRI), City of Scientific Research and Technological Applications (SRTA-City), New Borg Al-Arab City, Alexandria, 21934 Egypt; 3 Al Ezz Dekheila Steel Company, Alexandria, Egypt

**Keywords:** Nanoscience and technology, Physics

## Abstract

This work is an attempt to employ the electric arc furnace (EAF) slag as a by-product material to develop an alternative and environmentally friendly material for gamma-radiation protection applications such as in medical and industrial areas. For this purpose, different concentrations of micro-sized EAF slag (0, 20, 40, 60, 80, 100, 500, and 800 phr) were incorporated as fillers in the natural rubber (NR) matrix to produce the shielding composites. In addition, nano-sized EAF slag particles were prepared by using a high-energy ball milling technique to investigate the effect of particle size on the gamma-radiation shielding properties. The synthesized micro and nano EAF/NR composites were tested as protective materials against gamma-radiation by employing NaI(Tl) scintillation detector and standard radioactive point sources (^152^Eu, ^137^Cs, ^133^Ba, and ^60^Co). Different shielding parameters such as linear and mass attenuation coefficient, half value layer (HVL), tenth value layer, mean free path, effective atomic number (Z_eff_), and effective electron density (N_eff_) were determined to assess the radiation shielding capability of the EAF/NR composites. Furthermore, equivalent atomic number (Z_eq_) and the exposure buildup factor values for photon energy in the range from 0.015 to 15 MeV were also computed by Geometric Progression method. The experimental results of micro EAF/NR composites showed that at 121.78 keV, EAF0 composite (without EAF slag content) had the lowest μ value of 0.1695 cm^−1^, while the EAF800 composite (which was loaded with 800 phr of micro EAF slag) had the highest μ value of 0.2939 cm^−1^ at the same energy, which in turn decreases the HVL from 4.09 to 2.36 cm, respectively. Therefore, increasing the filler weight fractions of EAF slag in the NR matrix, increases the shielding properties of the composites. Moreover, the NR composite reinforced with 800 phr of nano EAF slag has better gamma-radiation shielding efficiency compared to that filled with 800 phr of micro EAF slag. The success of this work was to prepare a flexible, lightweight, low-cost, and lead-free material with better shielding capability.

## Introduction

The applications of ionizing radiation have spread in many fields of industry, agriculture, medicine, scientific research, and others. Despite the multiple benefits of this radiation, they may cause severe damage to humans and the environment if they are not handled with great care and in safe ways^1^. The risks of using ionizing radiation in an unsafe manner may outweigh its benefits. Thus, shielding materials must be provided to attenuate radiation and protect workers and other equipment from harmful radiation. In choosing a protective shielding material, some characteristics must be available in the selected materials, such as high density and high atomic number (Z), in addition to low cost and availability^2^. For this purpose, lead is the first option and the most used material to protect from X-rays and γ-rays. But the usage of pure lead has some drawbacks, including toxicity, heaviness, rigidity, and poor portability. These shortcomings have prompted scientists and researchers in the field of radiation protection to find new alternative materials for radiation shielding. These alternative materials must be highly efficient to protect against radiation and possess characteristics that can overcome the shortcomings of lead, such as being lightweight, easily portable, and flexible.

Polymer composites have been widely investigated as an alternative choice due to their unique properties such as reasonable cost, ease of the process, light weight, flexibility, and good mechanical strength^3^. Polymer composites doped with fillers of metal or metal oxides of high atomic numbers such as W, Bi, Ba, Gd, and Sn have attracted the attention of many scientists for future applications in radiation shielding. Furthermore, polymer composites can not only be utilized as shielding materials against X-rays and gamma-rays when injected with elements of high atomic number but can also be employed as neutron moderators due to their high content of hydrogen atoms^4^.

Natural and synthetic rubbers are polymers with elastic properties often used in daily applications^5^. When combined with conductive additives such as carbon black, carbon fiber, metal, or metal oxide, these rubber-conductive composite materials have broad uses in electromagnetic interference shielding^6^ and many other electronic and electrical applications^7^. Adding fillers to rubber aims to improve some of its physical properties and develop the rubber industry. The fillers injected into the rubber composites can be made of organic or inorganic materials with different chemical structures, shapes, sizes, concentrations, and characteristics. Carbon black, calcium carbonate, mica, aluminum hydroxide, silica, clay minerals, barites, a variety of oxides, montmorillonite, and metals are some examples of fillers^8^. Good distribution of fillers in the rubber composites helps to achieve optimum reinforcement.

Natural rubber (NR) composites have high flexibility, good compressibility, significant stretch ability and good electrical conductivity^9^. The usage of rubber composites as radiation shielding materials has also been explored by several researchers due to these benefits. El-Khatib et al.^10^ reported low-cost and effective composites based on natural rubber and filled with lead as promising materials for fabricating protecting clothes against gamma-radiation. In another research, different fillers, including BaCO_3_, Bi_2_O_3_ and BaSO_4_, were compared as X-rays and gamma-rays shielding fillers in NR-based composites. The results demonstrated that the NR/Bi_2_O_3_ composite was the potential candidate to be used as a wearable and flexible radiation protective material^11^. The thermal neutrons shielding performance of NR composites loaded with different amounts of boric acid (H_3_BO_3_) has also been investigated and the total macroscopic cross-section of 0.29 cm^−1^ was reported at 30 phr of H_3_BO_3_^12^. Furthermore, recycled rubber was also investigated as radiation protection material with appropriate additives^13^. As a theoretical study, the gamma radiation shielding features of ethylene propylene diene monomer (EPDM) rubber composites filled with 200 phr of different metal oxides (Al_2_O_3_, CuO, CdO, Gd_2_O_3_, or Bi_2_O_3_) were reported using Geant4 Monte Carlo simulation toolkit^14^.

Recently, the application of nanomaterials in numerous branches of technology and science has gained the attention of scientists. The use of nanoparticles as fillers in the polymer matrix is growing fast. Several studies showed how adding nanoparticles as fillers to the polymeric matrix improved the shielding effectiveness of polymer composites^15^. Fillers in nanoscale have outstanding physical and chemical properties compared to their bulk counterparts and increase the ability of composites to attenuate radiation^16^. This is because nano-sized fillers can disperse more uniformly and with less agglomeration inside the polymer matrix compared to micro-sized fillers^17–19^. Plangpleng et al.^20^ demonstrated that the NR filled with BaSO_4_ nanoparticles provided better gamma radiation shielding than the ordinary-sized BaSO_4_ due to the large surface to volume area of nanocomposites.

Electric arc furnace (EAF) slag is a by-product generated during the steel production process and contains some chemicals, including recycled steel scrap, coke, lime, and some metal oxides^21^. Approximately one ton of slag wastes is generated for three to four tons of stainless steel^22^. The principal components of slag used and studied to date include Ca-silicates, Ca-Al-ferrites, molten oxides of calcium, iron, magnesium, and manganese^23^. Slags are used daily in a broad range of applications, including final landfill cover material^24^, Portland cement manufacturing^25^, an agricultural fertilizer^26^, and mineral CO_2_ sequestration^27^. It has also been used in wastewater treatment^28^ and as an economical material for environmental remediation^29^. Moreover, iron slag has also been investigated as an aggregate replacement to enhance the γ-ray shielding properties of concrete^30^.

This work is an attempt to develop flexible, low-cost, and effective radiation protective composites based on natural rubber as a polymer matrix and filled with micro-size EAF slag with a concentration of 0, 20, 40, 60, 80, 100, 500, and 800 phr (parts per hundred parts of rubber). The effect of nano-sized EAF slag on the γ-ray shielding performance of NR composites at 800 phr is also examined. EAF slag nanoparticles is prepared by ball milling and charachterized by transmission electron microscope (TEM). Moreover, the cross-section morphologies of the synthesized NR composites were examined using a scanning electron microscope (SEM). The linear and mass attenuation coefficients, the half-value layer, tenth-value layer, and mean free path of the examined NR composites are experimentally determined at different gamma-ray energies ranging from 121.78 to 1408.01 keV by using NaI scintillation detector. Furthermore, essential shielding parameters, such as effective atomic number (Z_eff_), electron density (N_eff_), equivalent atomic number (Z_eq_) and the exposure buildup factors (EBF) of the investigated NR composites are theoretically computed to assist the shielding capability of the proposed NR composites.

## Materials and methods

### Materials

A commercial natural rubber (NR) supplied by the Transportation and Engineering Co., Egypt, with SMR-20 grade and specific gravity of 0.934 g/cm^3^, was used as a polymer matrix in this study. Other chemicals, including Sulphur, Zinc oxide, Stearic acid, and Paraffin oil, are utilized in a commercial-grade without being purified and are locally delivered from ADWIC Co. Egypt. Moreover, N220 (ISAF) carbon black (CB) purchased from Birla Carbon Egypt Co. was used in this work. EAF Slag supplied from EZZ Steel Co. Alexandria, Egypt, is used as filler in the rubber matrix. Table 1 displays the elemental analysis of EAF Slag powder using Energy Dispersive X-ray spectroscopy (EDX) analysis.

**Table 1:** EDX analysis for EAF slag powder.

Element	Mass %
C	2.53 ± 0.10
O	39.57 ± 0.43
Mg	1.39 ± 0.08
Al	1.59 ± 0.08
Si	5.47 ± 0.13
P	0.08 ± 0.03
S	0.07 ± 0.03
Cl	0.53 ± 0.04
K	0.07 ± 0.03
Ca	17.03 ± 0.22
Ti	0.20 ± 0.04
Mn	0.74 ± 0.08
Fe	30.73 ± 0.39

### Synthesis of EAF slag nanoparticles (EAFS NPs)

EAFS NPs prepared by high-energy ball milling (Fritsch Pulverisette 7, Germany) with a speed of 600 rounds per minute (rpm). The ball mill contains two vials of size 50 ml and an outer stainless steel body by inner grinding medium made of tungsten carbide. Tungsten carbide balls of various sizes with a total mass of 90 g and a diameter between 2 and 10 mm were employed, where the ball-to-powder weight ratio was set to be 5:1. Ethanol, a process control agent, was added during milling to prevent powder agglomeration.

### Synthesis of rubber composites sheets

The preparation of different formulations of micro EAF/NR and EAFS NPs/NR composites was carried out according to ASTM D 3185–99. All rubber ingredients were combined using the two-roll mill of rolling temperature control with the following dimensions: 460 mm outside diameter, 250 mm working distance, 16 rpm rolling speed, and a 1.4 gear ratio. After milling, the sample sheet was collected from the mill, cut, and put into a steel mold of 0.5 cm in thickness, 25 cm in width, and 20 cm in height and divided into four circles of 8.3 cm in diameter needed for the radiation protection test. The mold is placed between two layers of steel to obtain smooth surface samples. Then the rubber composite was vulcanized using a hot press at 143 ± 2 °C for 20 min with an applied pressure of 15 MPa. Table 2 displays the prepared EAF/NR composites along with their ingredients.

**Table 2:** EAF/NR composites ingredients.

Ingredients (phr)	Samples								
	EAF0	EAF20	EAF40	EAF60	EAF80	EAF100	EAF500	EAF800	Nano EAF800
Natural rubber (NR)	100	100	100	100	100	100	100	100	100
Carbon black (N220)	30	30	30	30	30	30	30	30	30
Zinc oxide	5	5	5	5	5	5	5	5	5
Paraffinic oil	10	10	10	10	10	10	10	10	10
6PPd	1	1	1	1	1	1	1	1	1
Stearic acid	2	2	2	2	2	2	2	2	2
CBS	2	2	2	2	2	2	2	2	2
Sulfur	2	2	2	2	2	2	2	2	2
EAF slag	0	20	40	60	80	100	500	800	800

### Structural analysis

The transmission electron microscope (TEM) (JEM 1400 Plus, JEOL, Japan) operating at 200 kV was used to measure the particle size of EAFS NPs. Furthermore, the cross-section morphologies of the synthesized NR composites were examined using a scanning electron microscope (SEM) (JSM-6010LV, JEOL). The specimens were coated with an ultrathin gold coating before SEM examination using low-vacuum sputtering coating equipment (JEOL-JFC-1100E). The SEM images were acquired at a voltage of 20 kV and 20,000× magnification.

### Gamma-ray acquisition setup

**Figure 1:**
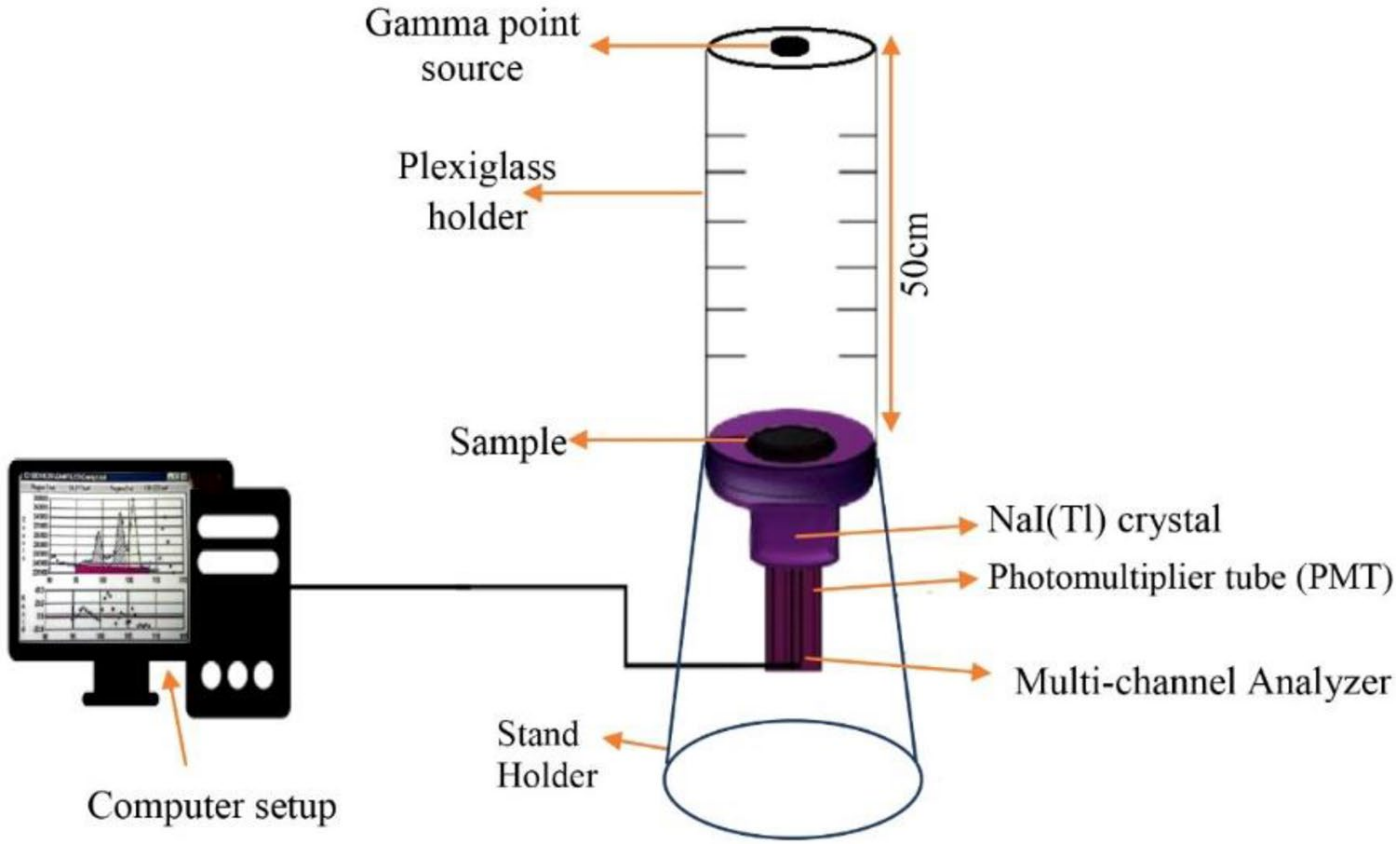
Experimental arrangement for gamma-ray measurement.

Gamma ray shielding properties of the investigated micro EAF/NR and EAFS NPs/NR composites were performed by using 3′′ × 3′′ NaI scintillation detector (Canberra Model 802), and has a resolution of 7.5% at the 662 keV. Four standard radioactive point sources, including ^60^Co, ^133^Ba, ^137^Cs, and ^152^Eu, were employed to measure the shielding parameters of the selected composites at different energies. These sources, along with their generated energies, emission probabilities, and half-life time are listed in Table 3^31^. The radioactive source was placed on a Plexiglas holder at a distance of 50.86 cm from the sample, which was placed on the detector surface. This distance between the detector’s surface and the radioactive source was chosen for several reasons, including obtaining a parallel beam, reducing the impact of dead time, and ignoring the effect of coincidence summing^32^. According to the sample thickness, the gamma spectra for all measurements were collected for enough time so that the statistical error in the net area under each peak was less than 1%. The Genie 2000 software was employed to analyze the obtained spectra, and the count rate was measured at a given energy and thickness for each prepared NR composite and then tabulated on an excel sheet. The experimental setup for the gamma-ray measurements is shown in Fig. 1.

**Table 3:** Photon energies, emission probabilities per decay and half-life time for all radioactive sources^31^.

Radioactive source	Photon energy (keV)	Emission probability %	Half life time (Days)
^60^Co	1173.23	99.90	1925.31
	1332.5	99.982	
^133^Ba	356.01	61.90	3847.91
^137^Cs	661.66	85.21	11,004.98
^152^Eu	121.78	28.40	4943.29
	244.69	7.49	
	344.28	26.60	
	778.90	12.96	
	964.13	14.00	
	1408.01	20.87

### Theoretical background

The experimental density ρ of each composite was measured at room temperature by employing Archimedes principle, according to ASTM D 792-9124^33^, using a well-calibrated single pan electrical balance (GR200, Japan) and an organic liquid of known density ρ_l_. The measured density is calculated using Eq. (1):

1 $$ \rho = \frac{{w_{a} }}{{W_{a} - W_{l} }} \times \rho_{l} $$

where w_a_ is the weight of the composite in air and w_l_ is the weight of composites in the organic liquid.

The lightweight and low density are the most advantages of polymer composite materials. The parameter which describes this characteristic is called heaviness which was calculated relative to the lead by Eq. (2)^34^:

2 $$\mathrm{Heaviness \% }=\frac{denisty\, of\, the\, composite}{denisty\, of\, lead} \times 100$$

When gamma-ray of appropriate energy passes through matter, it is subjected to attenuation due to the photoelectric effect, Compton scattering, and pair production interactions. As a result of these interactions, the intensity of the radiation is decreased exponentially as a function of the thickness of the absorbing material as described by Eq. (3)^35^:

3 $$I={I}_{0} {e}^{-\mu x}$$

where I_0_ and I are the incident and transmitted γ-ray intensities, respectively, x is the thickness of the absorbing medium, and μ (cm^−1^) is called the linear attenuation coefficient. μ is one of the most significant shielding parameters that depend on γ-ray energy and the absorber composition. The mass attenuation coefficient μ_m_ was then determined by simply dividing the estimated μ of a given composite by its density ρ as shown by Eq. (4)^15^:

4 $${\mu }_{m}=\frac{\mu }{\rho }$$

Other shielding parameters, such as half-value layer (HVL) and tenth-value layer (TVL), were calculated using Eqs. (5, 6), are parameters represent the absorber thicknesses needed to reduce the incident γ-ray intensity to 50% and 10% of its initial value, respectively^36^:

5 $$HVL= \frac{Ln 2}{\mu }$$

6 $$TVL=\frac{Ln 10}{\mu }$$

When describing the shielding characteristics of compounds or composites in terms of pure elements, the effective atomic number (Z_eff_), a useful photon interaction parameter that depends on photon energy, is utilized. Equation (7) is used to derive Z_eff_ values for the investigated EAF/NR composites^37^:

7 $${Z}_{eff}=\frac{{\sum }_{i}{f}_{i}{A}_{i}{\left({\mu }_{m}\right)}_{i}}{{\sum }_{j }{f}_{i }\frac{{A}_{j}}{{Z}_{j}}{\left({\mu }_{m}\right)}_{j}}$$

where f_i_, A_i_, and Z_i_ are the molar fraction, the atomic weight, and the atomic number of the ith constituent element in the composite material.

The effective electron density (N_eff_), expressed in electrons/g, is the quantity of electrons present in the composite material per unit mass, and it is calculated using Eq. (8)^38^:

8 $${N}_{eff}=\frac{{N}_{A}{Z}_{eff}}{\langle A\rangle }$$

where N_A_ is the Avogadro’s number and $\langle A\rangle ={\sum }_{i}{f}_{i}{A}_{i}$ is the average atomic mass of the composite material.

The exposure-buildup factor (EBF) must be taken into account in order to correct the attenuation calculations caused by the accumulation of secondary photons as a result of Compton scattering in designing an effective shielding material. The Geometric-Progression fitting method (GP) was used to calculate the EBF for the examined EAF/NR composites, and the calculations were carried out in accordance with the three steps listed below:

1. First, the equivalent-atomic number (Z_eq_), an energy dependent parameter that describes the characteristics of the selected EAF/NR composites in terms of their equivalent elements, was determined using the formula^39^:
  9 $${Z}_{eq}=\frac{{Z}_{1}\left(\mathrm{log}{R}_{2}-\mathrm{log}R\right)+{Z}_{2}\left(\mathrm{log}R-\mathrm{log}{R}_{1}\right) }{\mathrm{log}{R}_{2}-\mathrm{log}{R}_{1}}$$
  where R_1_ and R_2_ are the (μ_Comp_/μ_total_) ratios corresponding to the elements with atomic numbers Z_1_ and Z_2_ respectively, and R is the (μ_Comp_/μ_total_) ratio for the polymer selected at a specific energy, which lies between ratios R_1_ and R_2_.
2. The estimated Z_eq_ values of the EAF/NR composites were then used to interpolate GP fitting exposure buildup factor coefficients (b, c, a, X_K_, d) in the energy range 0.015–15 MeV using the interpolation formula (10)^40^:
  10 $$C=\frac{{C}_{1}\left(\mathrm{log}{Z}_{2}-\mathrm{log}{Z}_{eq}\right)+{C}_{2}\left(\mathrm{log}{Z}_{eq}-\mathrm{log}{Z}_{1}\right) }{\mathrm{log}{Z}_{2}-\mathrm{log}{Z}_{1}}$$
  where C_1_ and C_2_ are GP fitting parameters, acquired from ANSI/ANS-6.4.3 standard database^40^, corresponding to Z_1_ and Z_2_ between which Z_eq_ of the selected composite lies.
3. Finally, using the determined GP fitting parameters and the following equations, the EBF for the investigated EAF/NR composites was derived^41^:
  11 $$B\left(E,x\right)=1+\frac{b-1}{K-1} \left({K}^{x}-1\right) , K\ne 1$$
  and
  12 $$B\left(E,x\right)=1+\left(b-1\right)x , K=1$$
  where

13 $$ K\left( {E,x} \right) = cx^{a}  + d\frac{{{\text{tanh}}\left( {x/X_{K}  - 2} \right) - {\text{tanh}}\left( { - 2} \right)}}{{1 - {\text{tanh}}\left( { - 2} \right)}}{\text{for}}\,x \le 40\,{\text{mfp}} $$

where E is incident photon energy and x is the penetration depth in terms of mfp.

## Results and discussion

### Characterization

#### Transmission electron microscope (TEM)

**Figure 2:**
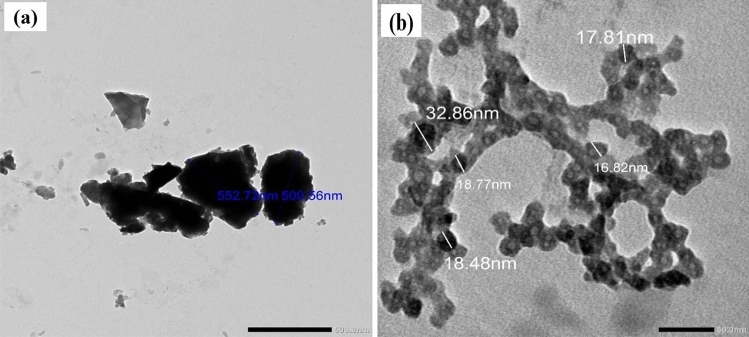
TEM images of (**a**) micro EAF slag and (**b**) nano EAF slag.

The TEM micrographs of micro EAF slag and nano EAF slag are shown in Fig. 2a,b, respectively. From Fig. 2a, it is clear that micro EAF slag particles have an irregular shape with varying particle sizes between 0.5 and 1 μm. On the other hand, Fig. 2b confirms the presence of EAF slag in nano dimension scale after ball milling with grains of spherical shape with a size distribution between 17 and 33 nm.

#### Scanning electron microscope (SEM)

**Figure 3:**
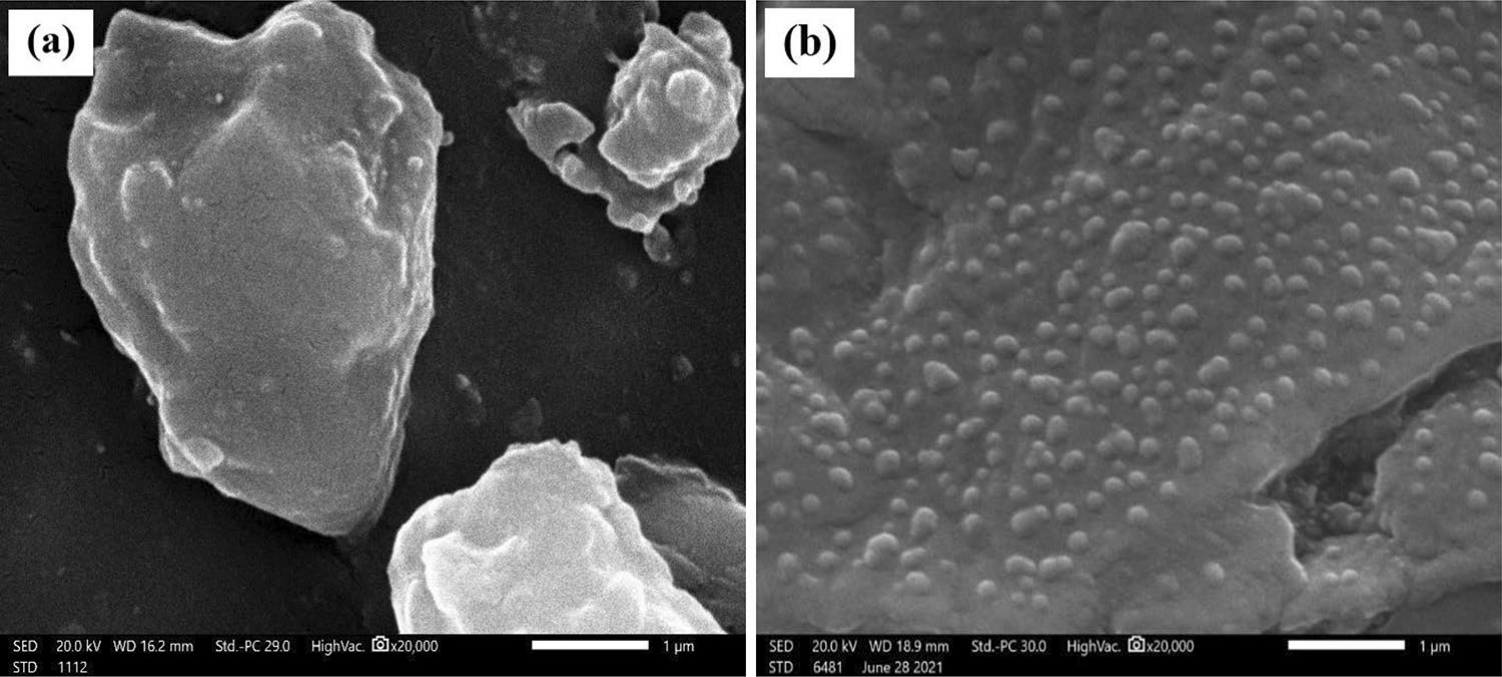
SEM images of (**a**) micro EAF800/NR and (**b**) nano EAF800/NR composites.

SEM was employed to examine the distribution of micro and nano EAF slag particles in the NR matrix. The SEM morphologies of the micro EAF800 and nano EAF800 composites are shown in Fig. 3a,b respectively. From Fig. 3a, it is obvious that micro EAF slag particles are not dispersed well with the NR matrix and there are gaps between bulky particles, and some of them are peeled away from the matrix providing voids for shielding. On the other hand, Fig. 3b reveals that nano EAF slag particles are homogeneously distributed and well embedded in the NR matrix which provides the interlocking structure for shielding.

### Gamma-ray shielding properties

#### Micro EAF/NR composites

The mass attenuation coefficients μ_m_ for the micro EAF/NR composites were experimentally estimated at different photon energies ranging from 121.78 to 1408.01 keV. The theoretical values of μ_m_ were also obtained from XCOM software^42^. The discrepancy Δ% between the experimental and theoretical values of μ_m_ was calculated by Eq. (14):

14 $$\Delta \%=\frac{{({\mu }_{m})}_{exp.}-{\left({\mu }_{m}\right)}_{XCOM} }{{({\mu }_{m})}_{XCOM}} \times 100$$

Table 4 lists the measured densities and mass attenuation coefficients for EAF/NR composites along with the theoretical μ_m_ values calculated from XCOM program and their relative deviations at different photon energies. Table 4 shows that, at different photon energies, the experimentally measured values of μ_m_ of the micro EAF/NR composites are fairly match to those predicted theoretically from XCOM software, where the discrepancy (Δ%) in the measured values of μ_m_ was estimated to be from ± 0.09% to ± 2.88%, which confirms a valid calibration of the experimental setup. It is evident from Table 4 that, μ values for all the proposed EAF/NR composites are dependent on the incident gamma-ray energy and the concentration of the added EAF slag.

**Table 4:** Measured values of density and mass attenuation coefficients for EAF/NR composites versus theoretical values calculated from XCOM program and their relative deviations at different photon energies.

Sample	Density	Energy (keV)	Mass attenuation coefficient (cm^2^ g^−1^)		Δ% (%)
			Experimental	Theoretical (XCOM)	
EAF0	1.066 ± 0.004	121.78	0.1589	0.1579	0.63
		244.69	0.1248	0.1248	0.00
		344.28	0.1118	0.1100	1.64
		356.01	0.1098	0.1086	1.10
		661.66	0.0851	0.0839	1.43
		778.9	0.0784	0.0779	0.64
		964.13	0.0704	0.0704	0.00
		1173.23	0.0652	0.0639	2.03
		1332.5	0.0600	0.0599	0.17
		1408.01	0.0586	0.0582	0.69
EAF20	1.130 ± 0.007	121.78	0.1620	0.1604	1.00
		244.69	0.1215	0.1238	− 1.86
		344.28	0.1106	0.1089	1.56
		356.01	0.1071	0.1074	− 0.28
		661.66	0.0822	0.0829	− 0.84
		778.9	0.0765	0.0770	− 0.65
		964.13	0.0692	0.0696	− 0.57
		1173.23	0.0628	0.0632	− 0.63
		1332.5	0.0586	0.0592	− 1.01
		1408.01	0.0581	0.0575	1.04
EAF40	1.172 ± 0.008	121.78	0.1576	0.1624	− 2.96
		244.69	0.1249	0.1229	1.63
		344.28	0.1066	0.1079	− 1.20
		356.01	0.1057	0.1065	− 0.75
		661.66	0.0805	0.0822	− 2.07
		778.9	0.0741	0.0763	− 2.88
		964.13	0.0683	0.0689	− 0.87
		1173.23	0.0617	0.0626	− 1.44
		1332.5	0.0594	0.0586	1.37
		1408.01	0.0576	0.0570	1.05
EAF60	1.208 ± 0.005	121.78	0.1658	0.1640	1.10
		244.69	0.1234	0.1223	0.90
		344.28	0.1084	0.1072	1.12
		356.01	0.1068	0.1058	0.95
		661.66	0.0816	0.0815	0.12
		778.9	0.0759	0.0757	0.26
		964.13	0.0681	0.0684	− 0.44
		1173.23	0.0627	0.0621	0.97
		1332.5	0.0572	0.0582	− 1.72
		1408.01	0.0572	0.0566	1.06
EAF80	1.259 ± 0.004	121.78	0.1614	0.1653	− 2.36
		244.69	0.1214	0.1218	− 0.33
		344.28	0.1049	0.1066	− 1.59
		356.01	0.1036	0.1052	− 1.52
		661.66	0.0809	0.0810	− 0.12
		778.9	0.0747	0.0752	− 0.66
		964.13	0.0674	0.0680	− 0.88
		1173.23	0.0620	0.0617	0.49
		1332.5	0.0581	0.0578	0.52
		1408.01	0.0554	0.0562	− 1.42
EAF100	1.277 ± 0.006	121.78	0.1675	0.1664	0.66
		244.69	0.1210	0.1213	− 0.25
		344.28	0.1061	0.1061	0.00
		356.01	0.1040	0.1047	− 0.67
		661.66	0.0815	0.0806	1.12
		778.9	0.0751	0.0748	0.40
		964.13	0.0658	0.0676	− 2.66
		1173.23	0.0624	0.0614	1.63
		1332.5	0.0585	0.0575	1.74
		1408.01	0.0559	0.0559	0.00
EAF500	1.564 ± 0.008	121.78	0.1733	0.1744	− 0.63
		244.69	0.1180	0.1181	− 0.08
		344.28	0.1016	0.1024	− 0.78
		356.01	0.1007	0.1010	− 0.30
		661.66	0.0782	0.0775	0.90
		778.9	0.0728	0.0719	1.25
		964.13	0.0656	0.0650	0.92
		1173.23	0.0585	0.0590	− 0.85
		1332.5	0.0557	0.0553	0.72
		1408.01	0.0538	0.0538	0.00
EAF800	1.662 ± 0.009	121.78	0.1768	0.1765	0.17
		244.69	0.1165	0.1172	− 0.60
		344.28	0.0993	0.1014	− 2.07
		356.01	0.0975	0.1000	− 2.50
		661.66	0.0769	0.0767	0.26
		778.9	0.0709	0.0712	− 0.42
		964.13	0.0646	0.0643	0.47
		1173.23	0.0571	0.0583	− 2.06
		1332.5	0.0532	0.0547	− 2.74
		1408.01	0.0523	0.0532	− 1.69

**Figure 4:**
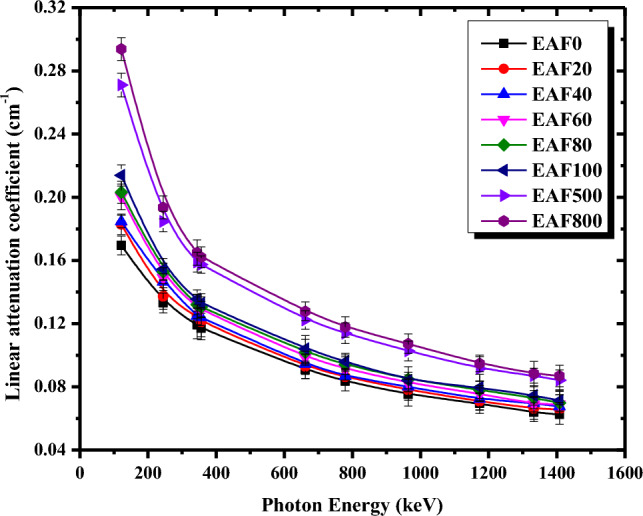
Linear attenuation coefficients of micro EAF/NR composites with different EAF slag concentrations as a function of photon energy.

The linear attenuation coefficient μ is a basic shielding parameter that can be investigated to assist the effect of adding different concentrations of EAF slag on the gamma-ray protective capability of the natural rubber matrix. The experimental values of μ for the current micro EAF/NR composites as a function of photon energy in the range between 121.78 and 1408.01 keV are plotted in Fig. 4. As can be seen from Fig. 4, loading the natural rubber with the EAF slag affects the shielding properties of the composites. It is clear from Fig. 4 that, the μ values increase regularly with increasing the concentrations of EAF slag from 20 to 800 phr in the natural rubber matrix and decrease sharply with increasing the incident photon energy. The increase in μ values with increasing the EAF slag content may be referred to the capability of EAF slag to attenuate gamma rays particularly in the low energy region. That is to say, μ increases by increasing the EAF content due to increase in the density of the composites (as shown in Table 4), since μ is a density dependent factor. However, the decrease in μ values with increasing the incident photon energy can be attributed to the three main gamma-ray interactions with matter: the photoelectric effect, Compton scattering, and pair production. The photoelectric effect is the dominant interaction at low photon energies, and its cross-section is inversely proportional to the photon energy E^3.5^; therefore, as expected, μ will decrease rapidly with increasing the photon energy. Moreover, at moderate energies between 661.7 and 1408 keV, the decrease in μ values with increasing photon energy becomes slightly significant. This is because, at these moderate photon energies, the Compton scattering becomes the dominant interaction with a cross-section probability that varies with E^−1^^43^.

**Figure 5:**
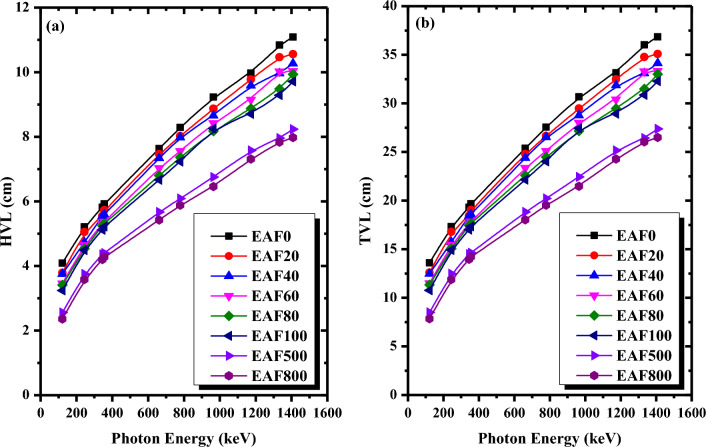
(**a**) HVL and (**b**) TVL of micro EAF/NR composites with different EAF slag concentrations as a function of photon energy.

HVL and TVL, frequently calculated shielding parameters, are used to evaluate the effectiveness of the shielding materials. HVL and TVL represent the absorber thicknesses needed to reduce the incident γ-ray intensity to 50% and 10% of its initial value, respectively. The better shielding materials have low HVL and TVL values. The variation of the HVL and TVL values of the selected micro EAF/NR composites as a function of photon energy is depicted in Fig. 5a,b, respectively. As can be seen from Fig. 5, the HVL and TVL values of the micro EAF/NR composites increase with increasing the gamma-ray energy while decreasing with increasing the content of the EAF slag. According to Fig. 5a, EAF800/NR composite has the lowest HVL values ranging from 2.35 to 7.97 cm, while EAF0/NR composite has the highest HVL values ranging from 4.09 to 11.08 cm. This suggests that the increase in the content of EAF slag reduces the HVL and TVL values leading to an improvement in the shielding effectiveness of the NR composites.

**Figure 6:**
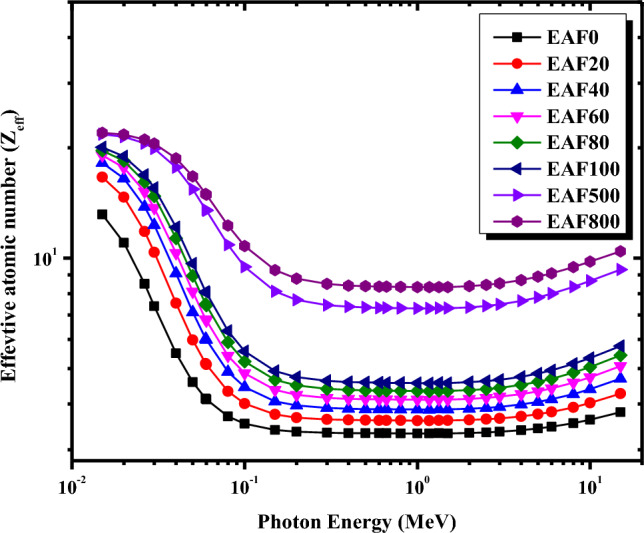
Variations of the effective atomic number for the micro EAF/NR composites with photon energy.

**Figure 7:**
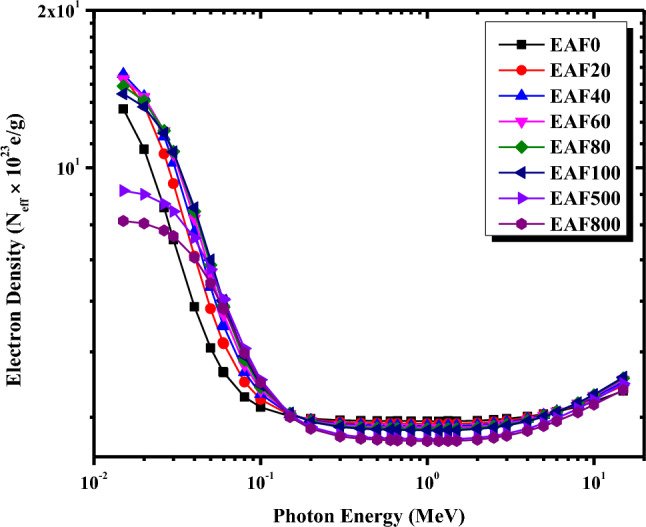
Variations of the effective electron density for the micro EAF/NR composites with photon energy.

The effective atomic number Z_eff_ and the effective electron density N_eff_ for the EAF/NR composites were computed theoretically in the energy range between 0.015 and 15 MeV and depicted in Figs. 6 and 7, respectively. Z_eff_ and N_eff_ were evaluated using μ_m_ values obtained from the XCOM database for each constituent element in the composite sample as described by Eqs. (7) and (8). It is evident from Figs. 6 and 7 that Z_eff_ and N_eff_ depend on the incident photon energy. Figure 6 verifies that in the energy range between 0.015 and 0.2 MeV, Z_eff_ for all the EAF/NR composites decreases quickly with the increase in the photon energy since the photoelectric effect is the predominant interaction in this energy range which varies inversely with E^3.5^. Further increase in the photon energy between 0.3 and 3 MeV, the Z_eff_ values are approximately constant for each composite due to the Compton scattering cross-section in this energy range. Due to the predominance of the pair production in the high energy region between 3 and 15 MeV, a slight increase in the Z_eff_ is noticed by increasing the energy of gamma-rays. It is also clear from Fig. 6 that Z_eff_ values increase apparently with increasing the concentrations of EAF slag from 20 to 800 phr in the natural rubber matrix at the same photon energy. The highest Z_eff_ is obtained for EAF800/NR composite and ranges from 21.97 to 10.44. In contrast, the lowest Z_eff_ is obtained for EAF0/NR and ranges from 13.14 to 3.80. Figure 7 shows N_eff_ is also a function in the incident gamma-ray energy. The dependence of N_eff_ on the incident photon energy can be discussed as in the Z_eff_ section. The highest N_eff_ is obtained for EAF800/NR composite and ranges from 21.97 to 10.44. In contrast, the lowest Z_eff_ is obtained for EAF0/NR composite and ranges from 13.14 to 3.80.

**Figure 8:**
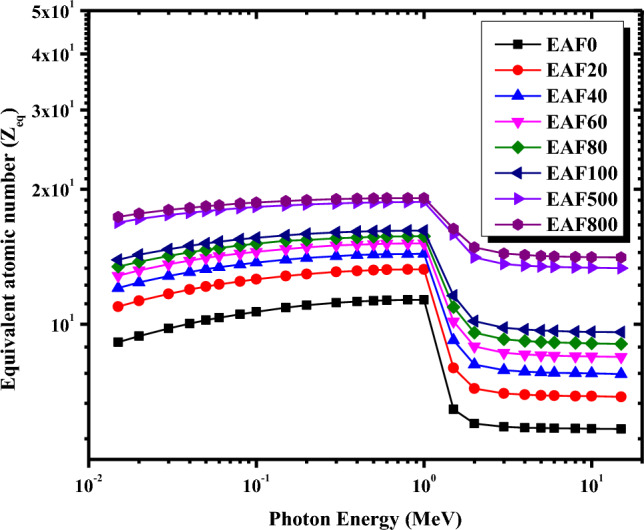
Variations of the equivalent atomic number for the micro EAF/NR composites with photon energy.

The interactions of gamma radiation at given energy with a material depend on the atomic number of the interacting medium. For this purpose, it is important to calculate the composite's equivalent atomic number (Z_eq_), which is synonymous with the elemental atomic number. The composite material with the higher equivalent atomic number is the best protective material. Figure 8 depicts the variation of Z_eq_ values for the micro EAF/NR composites versus gamma-ray energy. From Fig. 8, it is apparent that insertion of EAF slag in increasing amounts into the NR matrix causes the Z_eq_ to increase at the same gamma-ray energy. The highest Z_eq_ was found for the EAF800/NR composite, while the lowest Z_eq_ was for the EAF0/NR composite. Moreover, it is also obvious that the Z_eq_ increases gradually to reach its maximum value for all the EAF/NR composites at 1 MeV due to the Compton scattering process. Then, it falls rapidly as the γ-ray energy exceeds 1 MeV owing to the pair production process. Therefore, EAF800/NR composite has better shielding ability than other NR composites, which is consistent with the former results of linear attenuation coefficients.

**Figure 9:**
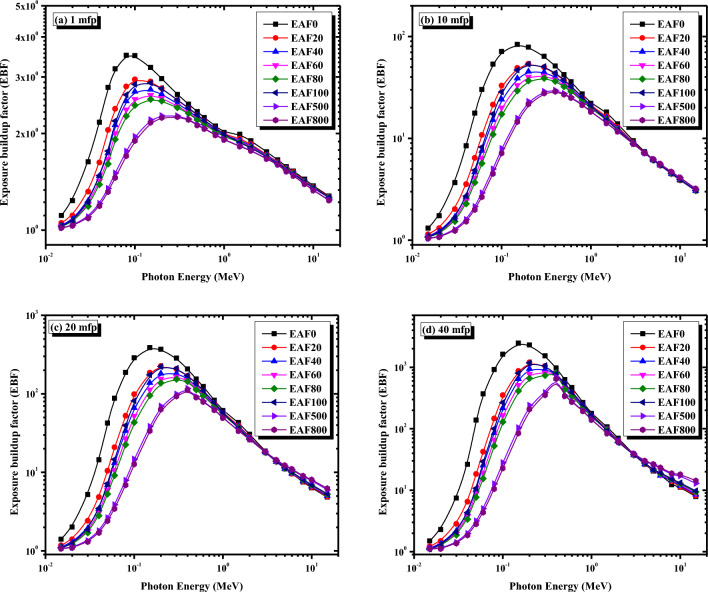
Variations of the exposure buildup factor with gamma ray energy for the micro EAF/NR composites at (**a**) 1 mfp, (**b**) 10 mfp, (**c**) 20 mfp, and (**d**) 40 mfp.

When selecting and developing an efficient protective material, the EBF must be taken into account to examine the effects of multiple gamma-ray scattering. Figure 9a–d depicts the variation of the EBF versus photon energy between 0.015 and 15 MeV for the micro EAF/NR composites at penetration depths 1, 10, 20, and 40 mfp. It is clear from Fig. 9 that the EBF values for all the EAF/NR composites are much higher at moderate gamma-ray energies between 0.08 and 0.5 MeV, where the Compton scattering process generates secondary photons and these photons are not totally removed. Still, they are prone to multiple scattering leading to a remarkable rise in the EBF values. On the other hand, at low and high gamma-ray energies, the EBF values are much smaller compared to moderate energies. This trend is due to the dominance of the photoelectric effect and pair production mechanisms, respectively, in which the photons are entirely absorbed or severely depleted their energies in low and high-energy regions. It is also clear from Fig. 9 that by increasing the content of the EAF slag in the NR matrix, the EBF values decrease, especially at low and moderate energies, and their maximums shift to larger energies. However, as the energy exceeded 1 MeV, increasing the concentration of EAF slag did not significantly affect the EBF values at the same penetration depth. Moreover, it is also revealed from Fig. 9a–d that the EBF values of the EAF/NR composites increase by increasing the penetration depths from 1 to 40 mfp. This can be attributed to the generation of multiple photons due to increased interactions of photons at large penetration depths.

#### Nano EAF/NR composite

**Figure 10:**
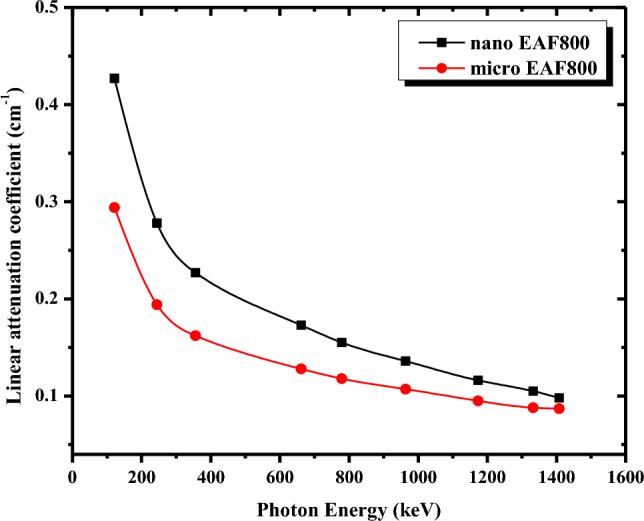
Comparison between linear attenuation coefficients of micro EAF800 and nano EAF800 composites as a function of photon energy.

The effect of nano-sized EAF slag on the γ-ray shielding performance of the NR matrix is also examined. The μ values as versus photon energy for nano EAF800 composite are compared to micro EAF800 composite as depicted in Fig. 10. The nano EAF800 composite (ρ = 1.) shows higher μ values than micro EAF800 at the same photon energy, indicating particle size’s impact on enhancing the shielding capability. The NR composite reinforced with 800 phr of nano EAF slag has better gamma-radiation shielding efficiency than that filled with 800 phr of micro EAF slag, which agrees with the findings published in the literature^44,45^. That is attributed to the homogenous dispersion of nano EAF slag particles within the NR matrix, as confirmed by the SEM micrographs, which increases the interaction probability between the incident photons and the nanoparticles.

**Figure 11:**
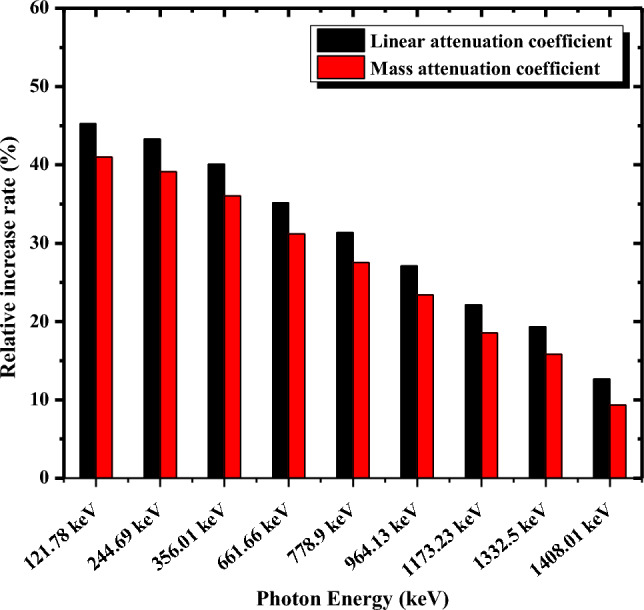
Relative increase rate in mass and linear attenuation coefficients between nano EAF800 and micro EAF800 versus photon energy.

The relative increase rate δ% in mass and linear attenuation coefficients between nano EAF800 and micro EAF800 are calculated by Eq. (15) and represented in Fig. 11.

15 $$\delta \%=\frac{{\mu }_{nano}- {\mu }_{micro} }{{\mu }_{micro}} \times 100$$

It is evident from Fig. 11 that the relative increase rate δ% decreases with increasing the photon energy, and the size effect becomes weak with the increase of photon energy. This trend is due to the dominance of Compton scattering cross section at higher energies which does not depend on Z of the constituent elements of the EAF slag filler. Therefore, the functional role of the EAF slag particle declines, and the impact of particle size diminishes compared to that at lower energies where the photoelectric effect dominates, and the absorption ability depends on the atomic number Z of the EAF slag particles. As a result, these nanoparticles play an important role in shielding radiation at low energies. In conclusion, nano EAF800 composite is a promising material for radiation protection applications spatially at low photon energies because it is an environmental (lead-free) material with low cost and lightweight compared with lead composites.

**Figure 12:**
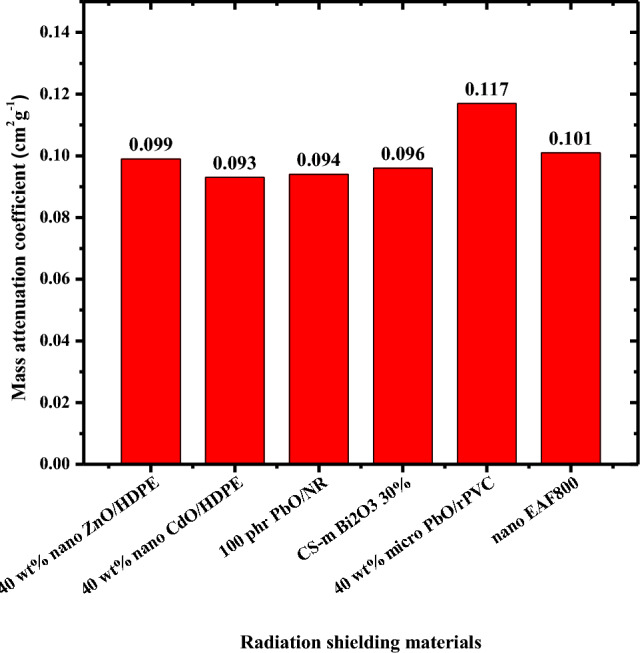
Reported mass attenuation coefficients of some composites in literature at 661.66 keV.

In order to assess the gamma-ray shielding ability of the composites introduced in this study, the mass attenuation coefficient of nano EAF800 composite is compared to other composites reported in literature (40 wt% nano ZnO/HDPE^44^, 40 wt% nano CdO/HDPE^15^, 100 phr PbO/WR/NR^10^, 40 wt% micro PbO/rPVC^45^, CS-m Bi2O3 30%^46^) at 661.66 keV as depicted in Fig. 12. As can be seen from Fig. 12, nano EAF800 composite shows a competing shielding ability with these previously published composites. Therefore, nano EAF-filled NR composite is a promising shielding material against gamma radiation.

## Conclusion

The current study aimed to prepare flexible, lightweight, and low-cost composites based on NR and filled with different concentrations of micro and nano EAF slag to be employed as comfortable clothing and gloves designed for workers in radiation facilities. From the obtained results, it can be concluded that the experimental values of μ_m_ for micro EAF/NR composites match well with those determined theoretically from the XCOM database. The measured values of the linear attenuation coefficients demonstrated their reliance on both the incident photon energy and the concentration of EAF slag. The increase in the content of the EAF slag increases the μ values and reduces the HVL and TVL values leading to an improvement in the shielding effectiveness of the NR composites. In addition, increasing the content of the EAF slag, decreases the EBF values, especially at low and moderate energies. The results also revealed that the particle size of the EAF slag plays a significant role in the shielding capability of the composite. The NR composite reinforced with 800 phr of nano EAF slag has better gamma-radiation shielding efficiency compared to that filled with 800 phr of micro EAF slag. That is attributed to the uniform dispersion of nano EAF slag particles within the NR matrix which increases the interaction probability between the incident photons and the nanoparticles. Thus, nano EAF800/NR composite is a promising alternative lead-free material for γ-ray shielding applications such as in medical and industrial areas.

## Data Availability

All data that support the findings of this study are included within the article.
